# Gut Microbial Changes in Diabetic db/db Mice and Recovery of Microbial Diversity upon Pirfenidone Treatment

**DOI:** 10.3390/microorganisms8091347

**Published:** 2020-09-03

**Authors:** Harinder Singh, Satoshi Miyamoto, Manjula Darshi, Manolito G. Torralba, Keehwan Kwon, Kumar Sharma, Rembert Pieper

**Affiliations:** 1 J. Craig Venter Institute, 9605 Medical Center Drive, Suite 150, Rockville, MD 20850, USA; kkwonus@gmail.com (K.K.); rpieper@jcvi.org (R.P.); 2 Department of Medicine, University of Texas Health, San Antonio, TX 77030, USA; s1miyamoto.research@gmail.com (S.M.); Darshi@uthscsa.edu (M.D.); SharmaK3@uthscsa.edu (K.S.); 3 J. Craig Venter Institute, 4120 Capricorn Lane, La Jolla, CA 92037, USA; MTorralba@jcvi.org

**Keywords:** gut microbiome, db/db mouse model, pirfenidone, cholecystokinin, type 2 diabetes, diabetic nephropathy, diabetic complications, kidney metabolism, metagenomics

## Abstract

The leptin receptor-deficient db/db mouse model is an accepted in vivo model to study obesity, type 2 diabetes, and diabetic kidney disease. Healthy gastrointestinal (GI) microbiota has been linked to weight loss, improved glycemic control, and physiological benefits. We investigated the effect of various drugs on the GI microbiota of db/db mice as compared to control db/m mice. Treatment with long-acting pirfenidone (PFD) increased gut microbial diversity in diabetic db/db mice. Firmicutes, the most abundant phylum in db/m mice, decreased significantly in abundance in db/db mice but showed increased abundance with long-acting PFD treatment. Several bacterial taxa, including *Lactobacillus* and some *Bacteroides*, were less abundant in db/db mice and more abundant in long-acting-PFD-treated db/db mice. Long-acting PFD treatment reduced the abundance of *Akkermansia muciniphila* (5%) as compared to db/db mice (~15%). We conclude that gut microbial dysbiosis observed in db/db mice was partially reversed by long-acting PFD treatment and hypothesize that PFD has beneficial effects, in part, via its influence on the gut microbial metabolite profile. In quantitatively assessing urine metabolites, we observed a high abundance of diabetic ketoacidosis biomarkers, including 3-hydroxybutyric acid and acetoacetic acid in db/db mice, which were less abundant in the long-acting-PFD-treated db/db mice.

## 1. Introduction

Type 2 diabetes (T2D) has emerged as a global public health problem. Epidemiological data show that the prevalence of this disease has increased significantly over the past several decades and continues to increase at alarming rates across the entire world (https://www.cdc.gov/diabetes/data/ statistics). People with diabetes spend 2.3 times more on medical expenditures as compared to people without diabetes, related primarily to T2D-associated chronic kidney disease (CKD) or diabetic kidney disease (DKD) [1,2]. T2D associated CKD is defined by persistently elevated urinary albumin excretion (UAE) ≥ 30 mg/g and a persistent reduction in the estimated glomerular filtration rate (eGFR) < 60 mL/min/1.73 m^2^, or both [3].

Gut microbiome profiles are known to be modulated by and to influence diabetes [4,5,6,7,8,9]. For example, changes in the gastrointestinal (GI) tract’s microbial composition were reported to increase gut permeability and result in the release of endotoxins such as lipopolysaccharides (LPS) into host circulation [10,11]. Inflammation that is initiated by bacterial LPS plays a role in the progression of DKD via toll-like receptor (TLR) innate immune response pathways [12]. CKD also affects the gut microbiota by disrupting the intestinal barrier with adverse outcomes that include uremic toxicity, infections, and inflammation [13,14,15,16]. Inflammation in rats with experimental uremia is associated with increased bacterial translocation from the gut into the blood circulation [17]. Microorganisms may ameliorate such outcomes: the administration of *Sporosarcina pasteurii* in uremic rats prolonged their life span and improved renal function [18]. GI tract microbiome signatures for cohorts of T2D patients include decreased abundances of Firmicutes along with increased abundances in the levels of beta-proteobacteria. It has been suggested that individuals with low bacterial richness are prone to developing pre-diabetes and T2D [19]. There have been at least two large-scale studies on gut microbiome dynamics related to T2D [6,7]. Qin et al. investigated the gut microbiome in 170 Chinese patients with T2D and 174 healthy controls and described T2D-associated microbial markers suggesting moderate dysbiosis with an increase in opportunistic pathogens and a decrease in butyrate producers in T2D patients [6,7]. More recently, correlations between *Akkermansia muciniphila* abundance and metabolic disorders were reported [20]. *A. muciniphila* is the most abundant mucin-degrading gut bacterium and protects the gut from pathogenic species by competitive exclusion [21]. In addition, *A. muciniphila* is thought to mediate glucose metabolism. Its abundance negatively correlates with inflammatory markers linked to metabolic disorders [20]. During fasting, malnutrition, and other nutrient deprivation conditions, *A. muciniphila* is more abundant in the gut due to its unique ability to use mucins as a nutrient source. Furthermore, human gut microbiota assembled gnotobiotic mice showed an increased abundance of *A. muciniphila* when fed with a fiber-free diet, which resulted in the absence of fiber-degrading species [22]. The abundance of *A. muciniphila* increased 80-fold after prebiotic intake of the bacteria in mice genetically predisposed to obesity with diet-induced leptin resistance [23]. T2D patients have a higher abundance of *A. muciniphila* as compared to non-diabetic controls in the gut microbiota, perhaps due to the intake of metformin [24].

*A. muciniphila* and *Bacteroides* are microbial organisms that are important to human health due to their production of short-chain fatty acids (SCFAs) in the colon. SCFA circulation in the human body is markedly influenced by the gut microbiome. Several SCFAs were lower in abundance in patients with a reduced eGFR, suggesting that diminished production of protective SCFAs aggravates disease progression, as shown for acute kidney disease [25,26]. Insulin resistance can be reversed in obese mice fed high-fat diets supplemented with SCFAs such as butyrate [27]. Butyrate-producing bacteria are negatively associated with biomarkers of inflammation [28], suggesting a modulatory role of certain gut bacteria in inflammation. In diabetic patients, inflammation due to the leakage of LPS through the GI tract mucosal barrier was shown to be chronic [29].

The db/db mouse is a model of interrelated pathologies such as obesity, diabetes, and dyslipidemia. The heterozygous mouse (db/m) does not display these pathologies, and the relevance of this model to study diabetic kidney disease was discovered [30]. Current treatments of diabetic nephropathy target the renin−angiotensin system (RAS). They include angiotensin-converting enzyme inhibitors (ACEIs) [31], angiotensin II receptor blockers (ARBs) [32,33], and tight glycemic control. In our previous randomized, placebo-controlled clinical study, we demonstrated that an orally administrated compound, pirfenidone (PFD), was able to reduce diabetic nephropathy and is effective in reducing glomerulosclerosis after the onset of established DKD in db/db mice [34,35]. PFD was found to inhibit TGF-β production which contributes to diabetic nephropathy and extracellular matrix deposition in animal models of lung and kidney disease [36,37,38]. In an open-label clinical study, patients taking pirfenidone had a 25% improvement in the estimated GFR (eGFR) rate of decline [39]. In this study, we have evaluated the effects of long- and short-acting PFD and the peptide hormone cholecystokinin (CCK) on the gut microbiome. CCK causes the release of digestive enzymes and bile from the pancreas and gallbladder and, therefore, also potentially affects gut microbiota in db/db mice. CCK is a well-known peptide hormone secreted from endocrine I cells of the duodenum and a regulator in the digestive tract and neurotransmitter in the nervous system. We have reported the renoprotective effects of CCK in diabetic mice [40]. To date, no clinical trials have examined CCK treatment effects in DKD patients, but CCK may be an ingredient of new treatments of DKD. PFD is an antifibrotic drug evaluated for the treatment of fibrotic diseases. It is FDA approved and has been used clinically for the treatment of idiopathic pulmonary fibrosis [41]. This state of research explains our rationale to survey the gut microbiome in the db/db model, including the effects of PFD and CCK. We hypothesize that changes in the gut microbiota in diabetic db/db mice (untreated and CKK- and PFD-treated) compared to lean db/m mice allow for insights into the role of gut microbes in diabetic nephropathy and potentially beneficial effects associated with PFD and CCK treatments.

## 2. Materials and Methods

### 2.1. Animal Experiments

All animal experiments were conducted in accordance with the Guide for Care and Use of Laboratory Animals (National Institutes of Health, Bethesda, MD, USA) and were approved by the local Institutional Animal Care and Use Committee at the University of California San Diego. The studies were performed in mice housed at 12:12 h light-dark cycle and free access to standard rodent chow obtained from Research Diet Inc. and tap water. Male db/db mice (BKS.Cg-Dock7^m^ +/+ Lepr^db^/J strain) and the corresponding heterozygous lean db/m mice were purchased from the Jackson Laboratory (Bar Harbor, ME). A total of 6 groups were included in this study: (1) db/m controls (*n* = 7); (2) db/db (*n* = 7), (3) db/db+short-acting PFD (*n* = 6), (4) db/db+long-acting PFD (*n* = 6), (5) db/db+low-dose CCK (*n* = 6), and (6) db/db+high-dose CCK (*n* = 6). At 16 weeks of age, db/db mice were provided with food pellets containing long- and short-acting PFD. In the CCK treatment groups, Alzet osmotic minipumps (Durect Corporation, Cupertino, CA, USA) were implanted subcutaneously in the backs of the mice. Mice in the db/db+low-dose CCK group and in the db/db+high-dose CCK group were continuously infused with CCK-8S (Bachem, Bubendorf, Switzerland) dissolved in 0.9% saline and given at a rate of 1 µg CCK-8S/kg/h and 5 µg CCK-8S/kg/h, respectively, over 4 weeks. Blood, cecal, and urine samples were collected at 20 weeks of age for analysis of the microbiota and metabolites. HbA1c was measured by the DCA Vantage Analyzer (Siemens, Malvern, PA, USA).

### 2.2. Cecum DNA Extraction

The DNA for 16S PCR was extracted from cecum samples using the MoBIO PowerSoil Purification DNA Isolation kit (Qiagen Inc. Valencia, CA, USA). Approximately 1 g of each cecum sample was resuspended in 800 µL of lysis buffer (1 M Tris-HCl, 2 mM EDTA, 1.2% Triton X-100) at 75 °C for 10 min. Lysed samples were cooled to room temperature, and 60 µL 200 mg/mL lysozyme and 5 µL RNase A were added. The lysates were incubated overnight at 37 °C. The DNA was extracted from the lysate using the manufacturer’s specifications and eluted in 100 µL of the final elution buffer [42].

### 2.3. 16S rDNA Analysis by MiSeq Sequencing

DNA extracted from cecum samples was amplified using primers that targeted the V1−V3 regions of the 16S rRNA gene [42]. These primers included the i5 and i7 adaptor sequences for Illumina MiSeq pyrosequencing as well as unique 8 bp indices incorporated onto both primers such that each sample receives its unique barcode pair. This method of incorporating the adaptors and index sequences onto the primers at the PCR stage provided a minimal loss of sequence data when compared to previous methods that would ligate the adaptors to every amplicon after amplification. This method also allows generating sequence reads, which are all in the same 5′−3′ orientation. Using approximately 100 ng of extracted DNA, the PCR was performed using a mixture consisting of 10 µL of 10× PCR Buffer, 3 µL of 50 mM MgCl_2_, 2 µL of 10 mM dNTPs, 1 µL of 10 µM F Primer N, and 0.5 µL Platinum Taq Polymerase (Life Technologies, Carlsbad, CA, USA) and the following cycling conditions: 95 °C for 5 min for an initial denaturing step; followed by 95 °C for 30 s, 57 °C for 30 s, 72 °C for 30 s for a total of 35 cycles; followed by a final extension step of 72 °C for 7 min then stored at 4 °C. Once the PCR for each sample was completed, the amplicons were purified using the QIAquick PCR purification kit (Qiagen Inc.), quantified fluorometrically using SYBR Gold (ThermoFisher Scientific, Waltham, MA, USA), normalized, and pooled in preparation for bridge amplification followed by Illumina MiSEQ sequencing using the dual index 2 × 300 bp format (Roche, Branford, CT, USA) following the manufacturer’s protocol.

### 2.4. Processing, Filtering, and Analysis of Sequence Reads

Operational taxonomic units (OTUs) were de novo generated from the raw Illumina sequence reads using the UPARSE pipeline [43]. Paired-end reads were trimmed of adapter, barcode, and primer sequences prior to assembly. Sequences of length less than 36 were discarded and leading/trailing low quality (below quality 3) bases were removed using Trimmomatic [44]. Sequences were subjected to a de-replication step and abundances were determined. Chimera filtering of the sequences occurred during the clustering step. The OTUs were generated using the usearch algorithm at the default 97% sequence similarity level [43]. We used the Wang classifier (method = wang) and bootstrapped using 100 iterations (iters = 100). We set mothur to report full taxonomies only for sequences where 80 or more of the 100 iterations are the same (cutoff = 80). Taxonomies were assigned to the OTUs with mothur [45] using version 123 of the SILVA 16S ribosomal RNA database [46] as the reference. Tables with OTUs and the corresponding taxonomy assignments were generated and used in subsequent analyses. The next step was to remove likely non-informative OTUs with an independent filtering process. Rare OTUs or taxa are strongly affected by MiSeq sequencing errors, and any statistical conclusions relying on them are typically unstable. Even in the univariate differential abundance analysis, the presence of such taxa increases the penalty from the multiple testing correction applied to the more abundant taxa. We used unbiased metadata-independent filtering at each level of the taxonomy by eliminating all features that did not pass these criteria. This included samples with less than 2000 reads and OTUs present in less than 10 samples. The phyloseq package version 1.16.2 in R package version 3.2.3 was used for the microbiota census data analysis [47,48]. The ordination analysis was performed using PCoA with the Bray–Curtis dissimilarity matrix. The alpha diversity was calculated using the Shannon index and the plots were generated using the ggplot2 package in R. We predicted the molecular functions present in the KO (KEGG Orthology) of the microbiota based on the OTU abundance and the OTU representative sequence data in Fasta format using the Piphillin web server [49].

### 2.5. Urine Metabolomics

Aliquots of frozen urine were thawed and analyzed for creatinine content using biochemical assay methods (i.e., 96-well based colorimetric assay kits, Cayman chemicals) before being processed for metabolomic analysis. Sample volumes equivalent to 0.5 to 1 µmol creatinine was used for GC–MS sample preparation. Urine samples, along with the internal standards, were diluted and the ketoacids were oxidized using O-(2,3,4,5,6-pentafluorobenzyl) hydroxylamine (PFBH), and samples were lyophilized overnight, followed by organic acid purification over silicic acid column chromatography. The enriched organic acids were dried under N_2_ and silylated at 60 °C. Around 1–2 µL of the derivatized sample was applied onto a 30 cm × 0.32 mm column (Agilent DB-5) in a gas chromatogram (Agilent 5890) at 80 °C and eluted with helium gas flow of 1 mL/min at 4 °C/min temperature gradient of 80 to 300 °C. The targeted analytes were detected by electron ionization, followed by multiple reaction monitoring using the Bruker Scion GCTQ. Each compound was identified using a quantifier and confirmed with 3–4 qualifiers. The final concentration of the metabolite in the urine samples are reported in µmol organic acid per mmol creatinine [50]. To ensure the reliability of the metabolite measurements, several quality controls were used. Within each batch, a pooled matrix sample master control (MC) samples was analyzed at the beginning, middle, and end of each batch. Metabolite concentrations of the MC must have within-batch CV of <10% or percent difference of <10%. Internal standards (structurally similar analog or stable labeled compound) were added to each sample at a constant concentration to facilitate the quantitation of target analytes. The main purpose of the analyses was to assess metabolic consequences of the db/db pathology in untreated and PFD and CCK treatment groups; we also attempted to link the data to SCFAs that are microbially produced in the gut and then pass the gut mucosal barrier.

### 2.6. Statistical Analyses for Gut Microbiota and Metabolites

To determine statistical changes in alpha diversity among different groups, we performed a Wilcoxon rank-sum test followed by Benjamini and Hochberg adjustment for *p*-value correction. The differential abundance test for the gut microbiota at a genus or species level was performed using the DESeq2 package version 1.12.3 in R was used [51] as suggested in the phyloseq protocol. The DESeq2 test uses a negative binomial model rather than simple proportion-based normalization or rarefaction to control for different sequencing depths, which may increase the power and also lower the false positive detection rate [52]. Default options of DESeq2 were used for its multiple testing adjustment applying Benjamini and Hochberg [53]. Permutational multivariate analysis of variance (PERMANOVA) calculations and analysis of similarities (ANOSIM) were performed using R VEGAN package version 2.4-2 [19]. For the classification of samples, we used the random forest algorithm version 4.6-12 implemented in R [20]. The metabolome data are analyzed using the MetaboAnalyst web server [54]. The samples were normalized by calculating the median for each sample and scaling the data using mean-centered and divided by the standard deviation of each variable. We performed t-test and fold change analysis between control db/m vs. diabetic db/db and four diabetic db/db treatment groups for metabolome data. The ggplot2 and ggsignify R packages were used to display the box plot and the significance level in stars based on *p*-value obtained by performing t-test between two different mice groups [48].

## 3. Results and Discussion

### 3.1. Metabolic Characteristics

**Figure 1:**
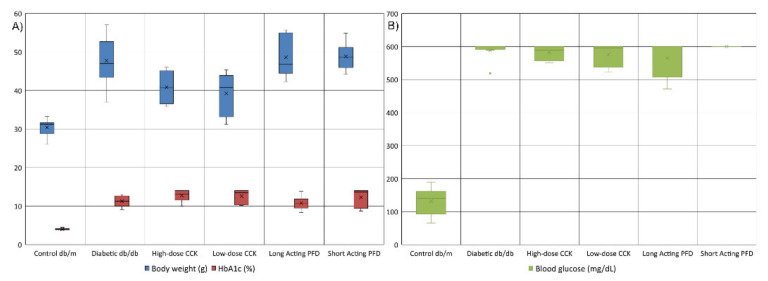
(**A**) The distribution of the HbA1c (%age) and body weight (g) of the control db/m (*n* = 7) and diabetic db/db (*n* = 6) mice as well as four treatment groups of diabetic db/db (*n* = 6 each group) (**B**) The distribution of glucose (mg/L) in various mice groups. High level of HbA1c correlated with chronic kidney disease (CKD). The x marker in the box plot represents the average value.

The db/db mice had significantly elevated body weights, HbA1c, and glucose levels compared to the db/m controls (Figure 1). High levels of HbA1C are associated with CKD [55]. For the control db/m mice, the average body weight, HbA1c, and glucose levels were 30.4 g, 3.99%, and 131.8 mg/dL, respectively. In the case of diabetic db/db mice, the average weight, HbA1c, and glucose levels increased to 47.7 g, 11.24%, and 588.5 mg/dL, respectively (Figure 1A). The db/db mice treated with long-acting PFD had slightly lower average HbA1c and glucose levels at 10.8% and 565.2 mg/dL, respectively, with negligible weight changes (Figure 1B). On the other hand, short-acting-PFD-treated db/db mice had higher HbA1c, weight, and glucose levels than db/db mice. Both high- and low-dose-CCK-treated db/db mice lost an average weight of 7.0 g and had small decreases in glucose levels but slightly higher levels of HbA1c as compared to db/db mice. Only CCK-treated mice significantly lost weight (*p*-value < 0.05) compared to db/db mice. Changes in other data types were not statistically significant.

### 3.2. Gut Microbiota Analysis

**Figure 2:**
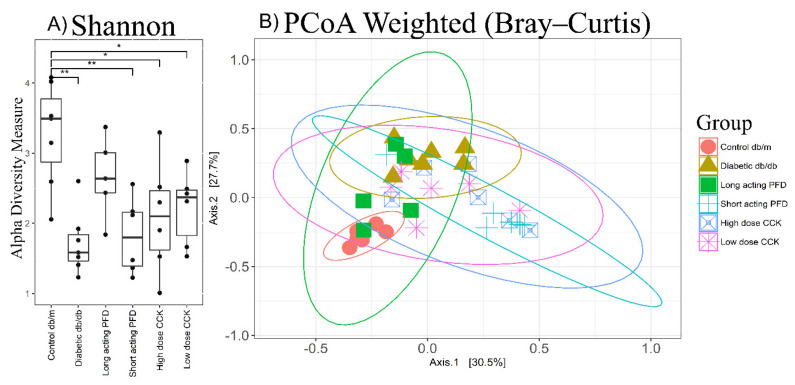
(**A**) Alpha diversity plot displaying the Shannon index for different groups with x-axis represents the Shannon index. Significance levels between two groups is indicated by stars (* 0.01 to 0.05, ** 0.001 to 0.01) and no comparison/stars mean no statistical significance was found between groups. High alpha diversity represents a healthy gut microbiome. (**B**) Principal coordinate analysis (PCoA) plot showing the control db/m clustering separate than db/db mice but overlapping with long-acting-PFD-treated db/db mice. The x- and y-axes represent the first and second components of the PCoA plot, respectively.

We compared the GI microbiota derived from control db/m mice, db/db mice, and four db/db mouse treatment groups using the 16S rRNA marker gene. A total of 688 OTUs were identified using the UPARSE pipeline, and the OTUs missing in more than 20% samples were removed from the analysis. A total of 456 OTUs were used to calculate the alpha diversity index and generate the PCoA plot. After merging all OTUs at the genus level, 119 genera were used for the remainder of the analyses. Out of 119 genera, only 16 genera represent the core microbiome of control db/m, diabetic db/db, and the four-treatment group (Supplementary Figure S4). Alpha diversity comparisons among the groups were performed using the Chao1 index (species richness) and Shannon or Shannon–Weaver index (species evenness) (Figure 2A and Supplementary Figure S1). The species richness is based on rare species and is a measurement of the number of species found in a community, and species evenness provides us abundance information of each species. Wilcoxon rank-sum tests using the Shannon index showed differences in the microbiota (*p*-value = 0.002) in control db/m vs. db/db groups and between three treatments groups compared to the db/m mouse group: 0.008 for short-acting PFD and 0.022 for high- and low-dose CCK (Supplementary Table T1). There were no significant differences (*p*-value 0.149) between the control db/m and long-acting-PFD-treated db/db mice. Using the Chao1 index, we did not observe significant differences for the control db/m group vs. the db/db group or the four treatment groups (*p*-value > 0.05) (Supplementary Table T1).

Using the classic multivariate ordination method based on Bray–Curtis dissimilarity and similarity matrices, principal coordinate analysis (PCoA) showed that the gut microbiota of control db/m and diabetic db/db can be discriminated based on the abundances of bacterial genera (weighted approach, Figure 2B) as well as based on the presence and absence of taxa (unweighted approach) (Supplementary Figure S2). In the treatment groups, control db/m mice clustered with a few of the long-acting-PFD-treated db/db mice (Figure 2B). The differences were validated using permutational multivariate analysis of variance (PERMANOVA R2 = 0.460, *p* = 0.001 and R2 = 0.280, *p* = 0.001 for weighted and unweighted approaches, respectively) and analysis of similarities (ANOSIM’s R = 0.856, *p* = 0.001 and R = 0.502, *p* = 0.001 for weighted and unweighted approaches, respectively). The treatment groups are clustered in between the control db/m and diabetic db/db mice groups in the PCoA plot (Figure 2B and Supplementary Figure S2). With the exception of the long-acting PFD treatment group (PERMANOVA R2 = 0.018, *p*-value = 0.057 and ANOSIM R = 0.250, *p*-value = 0.057 for weighted approach), the other three groups were significantly different from the control db/m microbiota (PERMANOVA *p*-value = 0.003 and ANOSIM *p*-value = 0.003). With the unweighted approach, long-acting PFD had the highest similarity with the control db/m group based on PERMANOVA test (R^2^ = 0.183, *p*-value = 0.016), but the outcome was different using the ANOSIM test (R = 0.148, *p*-value = 0.104). The other three treatment groups are statistically different from the control db/m microbiota. The ANOSIM test is more suitable for understanding the variation in beta diversity and not the beta diversity itself. We conclude that long-acting PFD partially restored microbiota from the db/db dysbiotic state towards the control db/m profile, excluding a few genera with very different abundances as compared to control db/m mice. We also compared the microbiota of diabetic db/db mice and the treatment groups using PERMANOVA and ANOSIM tests: the long-acting PFD group is different from the other treatment groups due to small R^2^/R ratios and a higher *p*-value (Table 1).

### 3.3. Differentially Abundant Genera

**Figure 3:**
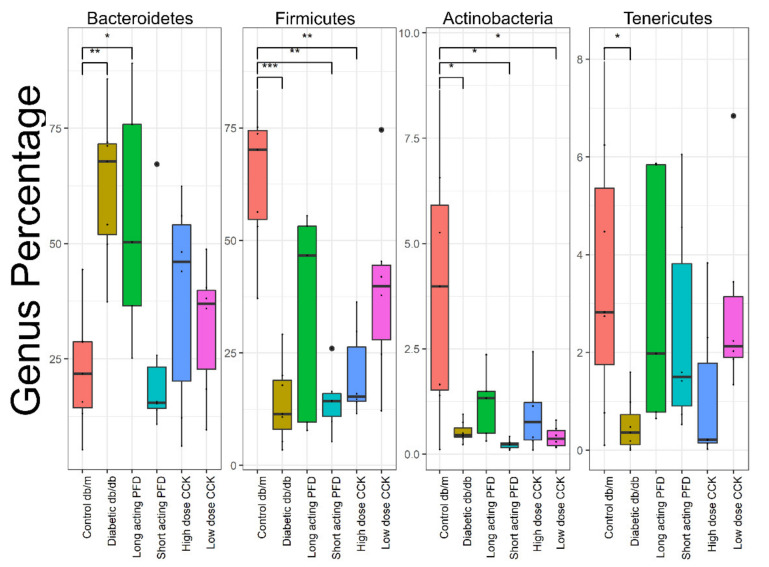
Phylum level comparison between control db/m, db/db, and four treatment groups. Significance levels between two groups is indicated by stars (* 0.01 to 0.05, ** 0.001 to 0.01, *** 0.0001 to 0.001) and no comparison/stars mean no statistical significance was found between groups.

Next, we used the DESeq2 package in R that uses a negative binomial model to determine the differentially abundant genera in the comparison of control db/m and db/db mice and with the four treatment groups. At the phylum level, the db/db mice had higher levels of Bacteroidetes and lower levels of Firmicutes, Actinobacteria, and Tenericutes. Mice treated with long-acting PFD had a higher abundance level of Firmicutes, Actinobacteria, and Tenericutes as compared to db/db mice, whereas short-acting PFD featured a lower abundance of Bacteroidetes as compared to db/db mice (Figure 3). Dominant SCFA producers belong to the phylum Firmicutes, and treatment with long-acting PFD indeed increased the abundance of Firmicutes towards the values for the control db/m mice [56].

**Figure 4:**
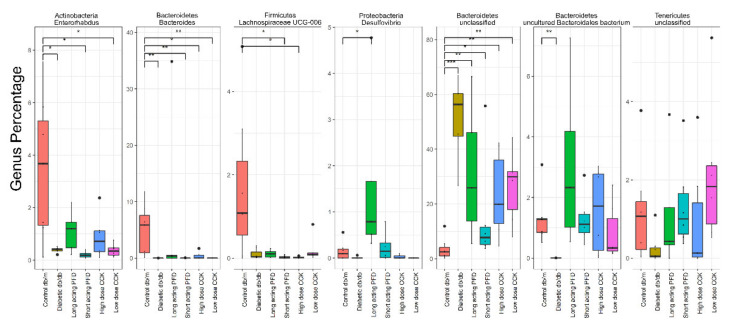
Genus level comparison between control db/m, db/db, and four treatment groups. The genus was selected using DESeq2 with FDR less than 0.05. Significance levels between two groups is indicated by stars (* 0.01 to 0.05, ** 0.001 to 0.01, *** 0.0001 to 0.001) and no comparison/stars mean no statistical significance was found between groups.

At the genus level, we observed lower abundance of *Bacteroides*, *Lachnospiraceae*, *Enterorhabdus*, unclassified Tenericutes and high abundance of unclassified Bacteroidetes in db/db mice compared to db/m mice. The taxonomic categories of *Bacteroides* and Bacteroidales had lower abundance in db/db mice, whereas unclassified Bacteroidetes were significantly higher in db/db mice as compared to db/m mice (Figure 4). Some species in the *Bacteroides* genus are mutualistic bacteria participating in the production of propionate and acetate [57]. Lachnospiraceae are responsible for the production of the important SCFA butyrate [58]. The UCG-006 category of Lachnospiraceae were by far the most abundant taxon in the db/m group. Members of the genus *Desulfovibrio* harbor virulence factors and can cause chronic inflammatory disorders such as IBD [59]. This genus was most abundant in the long-acting PFD group.

**Figure 5:**
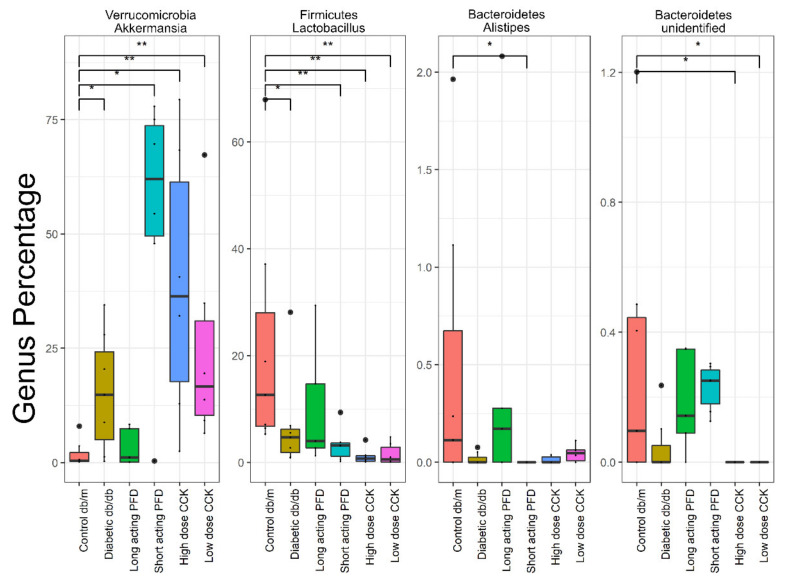
Selected genera with abundance levels comparable in long-acting PFD and control db/m cases obtained with DESeq2 with FDR less than 0.05. Significance levels between two groups is indicated by stars (* 0.01 to 0.05, ** 0.001 to 0.01) and no comparison/stars mean no statistical significance was found between groups.

In the case of db/db mice treated with long-acting PFD, *Akkermansia*, *Lactobacillus*, *Alistipes*, and unidentified Bacteroidetes abundances resembled those of control db/m mice more than those of diabetic db/db mice (Figure 5). A single species of the genus *Akkermansia* has been cultured, *A. muciniphila,* a mucin-degrading bacterium. *A. muciniphila* strengthens intestinal epithelial cell integrity and triggers cross-talk with the host by degrading the colonic mucin layer [60]. Its abundance in the gut is inversely correlated with the onset of inflammation during obesity [60]. Interestingly, db/db mice treated with short-acting PFD and, to some extent, those of the high-dose CCK group revealed a much higher abundance of *A. muciniphila* as compared to db/m mice and those of the long-acting-PFD-treated group. The average weights of the mice part of the long-acting and short-acting PFD groups as well as those of the db/db mice were similar, with approximately 48 g, and 15% lower than for high-dose CCK mice. Thus, there was no direct correlation between *A. muciniphila* abundance and the weight of mice.

### 3.4. Metabolite Changes Resulting from the Drug Treatment of Diabetic db/db Mice

**Figure 6:**
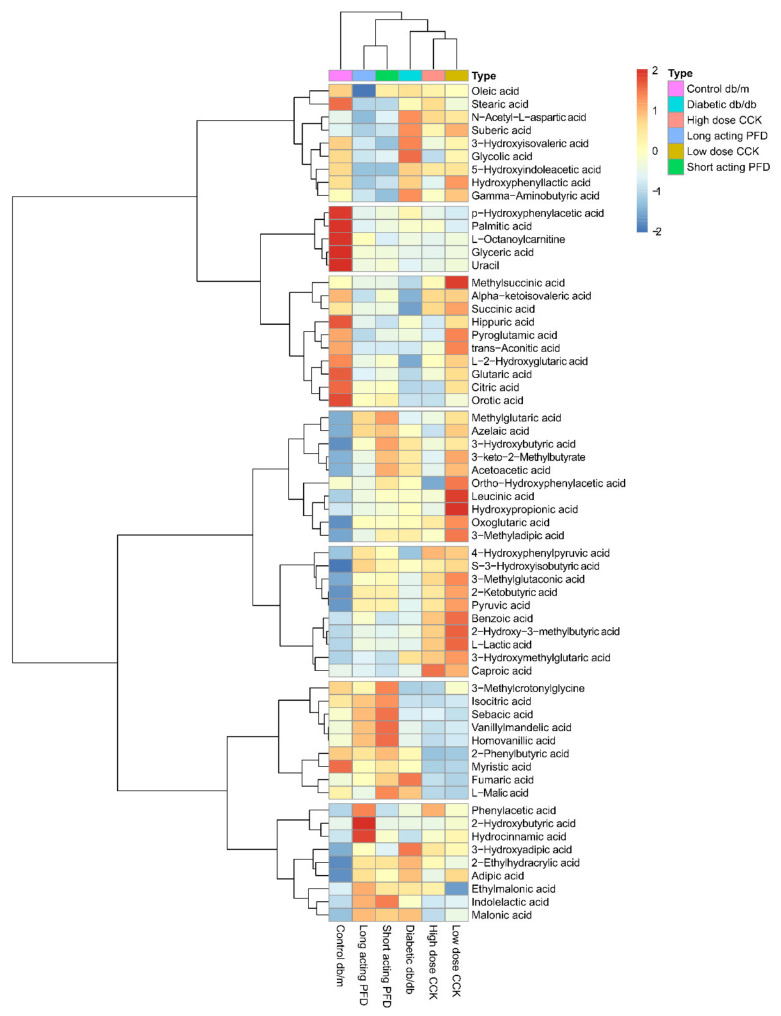
The heatmap display of the abundance of 62 metabolites comparing control db/m, diabetic db/db, and the four treatment groups along with clustering of mice groups and the metabolites.

We measured the abundances of 62 metabolites in urine, expecting data on kidney filtration and excretion products to be physiologically linked to renal functions in control db/m, diabetic db/db, and the four db/db mouse drug treatment groups. Metabolite abundance patterns clustered db/m mice in a defined space representing healthy kidneys. This was in good agreement with data on animal weight, blood glucose, and HbA1c levels. The db/db mice treated with long-acting and short-acting PFD clustered separately from db/db mice and CCK treatment groups (Figure 6; Supplementary Figure S3).

**Figure 7:**
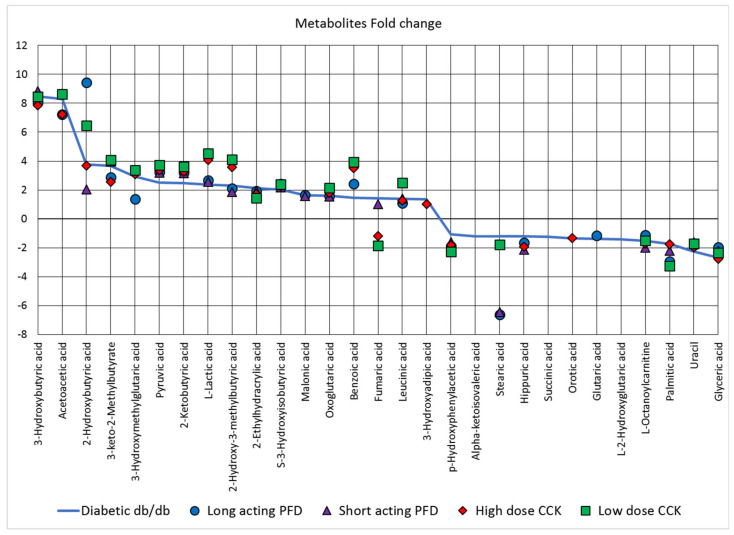
The log fold change of 29 metabolites differentially abundant in diabetic db/db mice (shown as a solid blue line) as compared to the control db/m mice (baseline at zero on the y-axis). The metabolites above the baseline at zero are more abundant, whereas metabolites below the baseline at zero are less abundant in db/db mice as compared to control db/m mice. The symbols represent the log fold change of the respected four treated mice groups in comparison with the control db/m mice.

Differentially abundant metabolites were analyzed with the MetaboAnalyst server. In Figure 7, the solid blue line represents the factor by which metabolite changes are observed in diabetic db/db mice as compared to control db/m mice. The metabolites above the baseline at zero are more abundant, whereas the metabolites below the baseline at zero are less abundant in db/db mice as compared to control db/m mice, while the four treatment groups are represented by their respective symbols. The most abundant metabolites are 3-hydroxybutyric acid and acetoacetic acid, ketone bodies that are markers of diabetic ketoacidosis. 2-Hydroxybutyric acid is a marker of insulin resistance and glucose regulation, possibly due to increased oxidative stress. Detection of 3-keto-2-methylbutyrate suggests a beta-ketothiolase deficiency, a disorder of isoleucine catabolism. 3-Hydroxymethylglutaric acid can accumulate due to reduced activity of intramitochondrial 3-hydroxy-3-methylglutaryl-CoA lyase. Pyruvic acid accumulates when its oxidation is compromised due to thiamine deficiency, thus affecting citrate cycle activity. 2-Ketobutyric acid is central to the metabolism of many amino acids and can be converted into propionyl-CoA, which is subsequently converted into succinyl-CoA (both SCFAs). Succinyl-CoA is a substrate of the citric acid cycle. Glyceric acid derivatives are important biochemical intermediates for glycolysis and required for the biosynthesis of some amino acids (e.g., Ser, Cys, Gly). Uracil, a nucleobase, is required for the production of RNA. Palmitic acid is a long-chain fatty acid produced during fatty acid synthesis. In summary, some quantified metabolites are markers of kidney malfunction, while others are intermediates of lipid metabolism without a biomarker role.

Treatment with long-acting PFD resulted in more moderate abundance changes in 3-hydroxybutyric acid, acetoacetic acid, 3-keto-2-methylbutyrate, 3-hydroxymethylglutaric acid, L-octanoylcarnitine, and glyceric acid compared to control db/m mice. The opposite trend was observed for 2-hydroxybutyric acid, benzoic acid, stearic acid, and palmitic acid. SCFAs that are of interest in healthy gut physiology (acetate, propionate, and butyrate) were not profiled in our experiments, but a few of their derivatives were. Derivates of butyric acid were less abundant in the urine of mice in the long-acting PFD group as compared to diabetic db/db mice. Other derivatives had higher abundances (Supplementary Table T2). Clear links between the predicted release of SCFAs into the circulatory system (based on gut microbial profiles) and their excretion into urine were not apparent. However, the more moderate abundance changes of 3-keto-2-methylbutyrate and 3-hydroxymethylglutaric acid that were observed for the long-acting PFD treatment group, compared to db/db mice, tentatively suggest higher activity of the two enzymes metabolizing these toxic compounds in renal tissue. Overall, none of the drug treatments drove metabolite abundances consistently into a range comparable to those of control db/m mice (Figure 6). Due to the small sample sizes (*n* = 6, 7), we did not perform correlation analyses for datasets derived from the gut microbiome and urinary metabolites.

**Figure 8:**
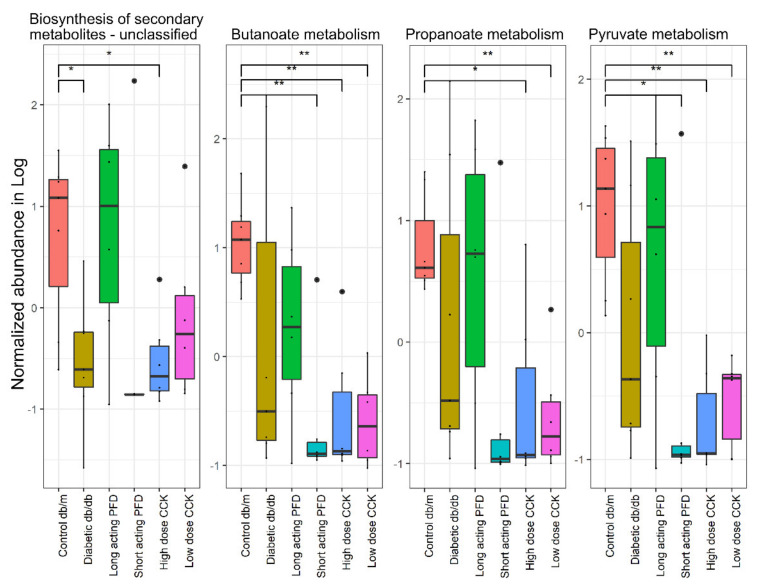
The molecular functions were predicted using the Piphillin web server. Their relative abundance in control db/m, diabetic db/db and the four treatment groups. Significance levels between two groups is indicated by stars (* = 0.01 to 0.05, ** = > 0.001 to 0.01) and no comparison/stars mean no statistical significance was found between groups.

The molecular functions present in the KO (KEGG Orthology) were inferred using the Piphillin web server and mapped to their respective KEGG pathways. One-way ANOVA was used to determine differentially abundant pathways comparing control db/m, diabetic db/db, and all four treatment groups. We observed 192 pathways with different abundances of molecular functions among the mouse groups with an FDR less than 0.05 (Supplementary Table T2 and Figure S5). The molecular functions of butanoate, propanoate, and pyruvate metabolism pathways (SCFA pathways) were downregulated in diabetic db/db mice as compared to control db/m mice. Apart from SCFA pathways, various molecular functions in pathways for secondary metabolite biosynthesis were also downregulated in diabetic db/db mice (Figure 8). Among the four treatment groups, only the group treated with long-acting PFD showed an increase in SCFAs production and biosynthesis of secondary metabolites, in further support of our hypothesis that long-acting-PFD-treated mice featured a recovery of gut microbiota diversity. These encouraging in silico results should be corroborated with evidence from deep metagenomic sequencing and SCFA quantitative measurements in the future.

## 4. Discussion

We identified strong changes in the gut microbiota of db/db mice compared to control db/m mice. Overall, microbial diversity decreased in db/db mice with highly abundant Bacteroidetes and lower abundances of Firmicutes, Actinobacteria, and Tenericutes. Several abundant taxa in the control db/m mice that decreased when compared to the db/db mice are *Bacteroides, Lachnospiraceae*, uncultured Bacteroidales, and *Lactobacillus*. Some species belonging to these genera break down complex carbohydrates via fermentative pathways and produce SCFAs such as acetate, propionate, and butyrate. They also assist in maintaining the mucosal membrane barrier function and the intestinal immune system. The producing microbes have mutualistic relationships with the host, protecting from the invasion of pathogens and via other physiological benefits [61]. Their absence or diminished abundance provides a chance for pathogens to colonize and thrive in the host’s gut. Another important genus is *Akkermansia,* which has one well-characterized species, *A. muciniphila*. While it was reported that obese mice ob/ob (C57BL/6) have a low abundance of *A. muciniphila* [62], we observed that db/db mice featured a higher abundance of *A. muciniphila* in the cecum. This observation does not correlate with weight gain in db/db mice. It was shown that higher abundances of *A. muciniphila* control and reverse high-fat diet-induced metabolic disorders [62]. The average abundance of *A. muciniphila* in healthy people ranges from 1% to 5% [63]. In our study, the treatment of db/db mice with long-acting PFD resulted in a lower abundance of *A. muciniphila*, resembling the data for control mice. We hypothesize that the gut microbial diversity recovers after treatment with long-acting PFD and normalizes the *A. muciniphila* abundance levels. We assume that there is a cause–effect relationship. The treatment with short-acting PFD and a high dose of CCK resulted in a significantly higher abundance of *A. muciniphila* as compared to the db/db mice. The *Lactobacillus* and Bacteroidetes abundances also changed. *Lactobacillus*, a genus with specific strains that are used in probiotics, *Alistipes*, and a few genera of Bacteroidetes increased quantitatively in db/db mice treated with long-acting PFD as compared to the other treatment groups. Alpha and beta diversity analyses suggest that gut microbial diversity and abundance levels improved with long-acting PFD as compared to other treatment groups.

*A. muciniphila* and other commensal microbes play important roles in human health via their production of metabolites, including SCFAs, that benefit gut mucosal integrity. The abundance of *A. muciniphila* depends upon factors such as diets, therapeutic drug intake, and health status. In the absence of fiber-degrading species, *A. muciniphila* abundance rapidly increases in the gut [22]. We also observed that the abundance of *A. muciniphila* directly correlates with that of the phylum Firmicutes. The latter are largely responsible for the degradation of dietary fibers. One study in China reported higher levels of *A. muciniphila* in T2D patients as compared to healthy people, possibly due to the intake of the drug metformin [7,64]. Our study suggests that a low abundance of Firmicutes paralleled the high abundance of *A. muciniphila* and treatment with long-acting PFD increased Firmicutes abundance and decreased *A. muciniphila* abundance, which assisted in improving the gut microbiome profile of db/db mice. In our study, quantities of 61 urinary metabolites normalized upon treatment with long-acting PFD (with a lower fold change compared to diabetic db/db mice). The markers of diabetic ketoacidosis, 3-hydroxybutyric acid and acetoacetic acid, decreased in abundance with the long-acting PFD treatment, suggesting improvement in renal functions. We acknowledge the challenge to mechanistically associate data on acidic metabolites in urine to similar metabolites (i.e., SCFAs which we did not directly measure in cecal specimens) that have known benefits in the gut mucosa. It is of interest to gain further insights into the metabolism of SCFAs absorbed in the colon, passing through and utilized in the liver and peripheral blood prior to excretion of metabolic derivatives via the kidneys. This will require comprehensive metabolic experiments with time courses and multiple sampling sites. The focus would be long-acting PFD because this treatment course has shown the most promising data to date. We hypothesize that long-acting PFD not only has anti-fibrotic effects but, at least in the db/db mouse model, a normalizing effect on the dysbiotic gut microbiome, either directly or via stabilization of renal functions. Direct normalization may also involve regeneration of physiologically normal gut permeability that we intend to investigate measuring transepithelial electric resistance of GI mucosal tissues from murine models, including analysis for all db/db treatment groups [65]. Overall, long-acting PFD treatment helps in the normalization of biomarkers of diabetic ketoacidosis as well as the gut microbiota profile in the db/db mice model.

## Figures and Tables

**Table 1:** PERMANOVA and ANOSIM test results between control db/m and diabetic db/db as well as four treatment groups using weighted and unweighted approaches with the Bray–Curtis distance matrix.

	Control db/m				Diabetic db/db			
	Weighted		Unweighted		Weighted		Unweighted	
PERMANOVA	R2	*p*-Value	R2	*p*-Value	R2	*p*-Value	R2	*p*-Value
Diabetic db/db	0.46	0.001	0.28	0.001	-	-	-	-
Long-acting PFD	0.02	0.057	0.18	0.016	0.20	0.020	0.19	0.01
Short-acting PFD	0.38	0.003	0.21	0.003	0.43	0.010	0.18	0.004
High-dose CCK	0.35	0.003	0.22	0.005	0.30	0.003	0.19	0.003
Low-dose CCK	0.32	0.003	0.3	0.003	0.31	0.003	0.21	0.005
ANOSI	R	*p*-Value	R	*p*-Value	R	*p*-Value	R	*p*-Value
Diabetic db/db	0.856	0.001	0.502	0.001	-	-	-	-
Long-acting PFD	0.25	0.057	0.148	0.104	0.29	0.19	0.257	0.033
Short-acting PFD	0.65	0.003	0.24	0.014	0.61	0.007	0.2	0.026
High-dose CCK	0.68	0.003	0.266	0.011	0.414	0.005	0.312	0.006
Low-dose CCK	0.5	0.003	0.43	0.004	0.44	0.003	0.289	0.009

## Data Availability

The 16s rRNA data are available on the Bio project number: PRJNA548916.

## Supplementary Materials

The following are available online at https://www.mdpi.com/2076-2607/8/9/1347/s1. Figure S1. The alpha diversity in terms of Chao1 comparison between diabetic db/db mice and control mice as well as the four treated control db/db mice with various drugs. Figure S2. Unweighted PCoA plot for all the groups along with 95% confidence interval. Figure S3. PCA plot with ellipse denotes 95% confidence interval. Figure S4. Core microbiome of control db/m, diabetic db/db and four treatment group. Figure S5. Their relative abundance of metabolites in control db/m, diabetic db/db and the four treatment groups. Table T1. Alpha diversity with Shannon and Chao1 diversity index differences using Wilcoxon test of the between control db/m and diabetic db/db and the four treated groups. Table T2. Log fold change of various metabolites in diabetic db/db mice and four treated groups in comparison with control db/m mice.
