# Supplementary material for: Gut Microbial Changes in Diabetic db/db Mice and Recovery of Microbial Diversity upon Pirfenidone Treatment
Source: Microorganisms. 2020 Sep 3;8(9):1347. doi: 10.3390/microorganisms8091347 (PMC7564638; doi:10.3390/microorganisms8091347)
Supplement: Supplementary file 1 [file microorganisms-08-01347-s001.pdf]

Supplementary Material

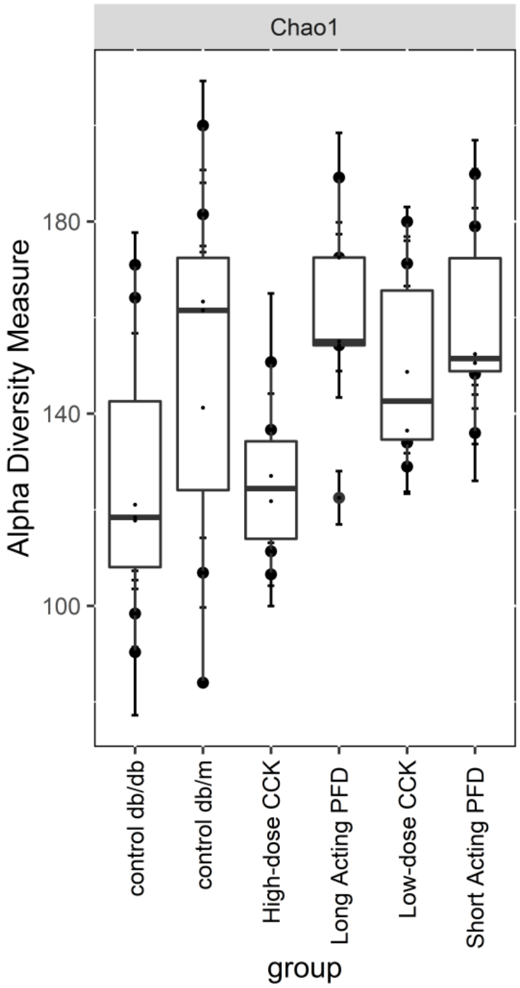

Supplementary Figure S1: The alpha diversity in terms of Chao1 comparison between diabetic db/db mice and control mice as well as the four treated control db/db mice with various drugs.

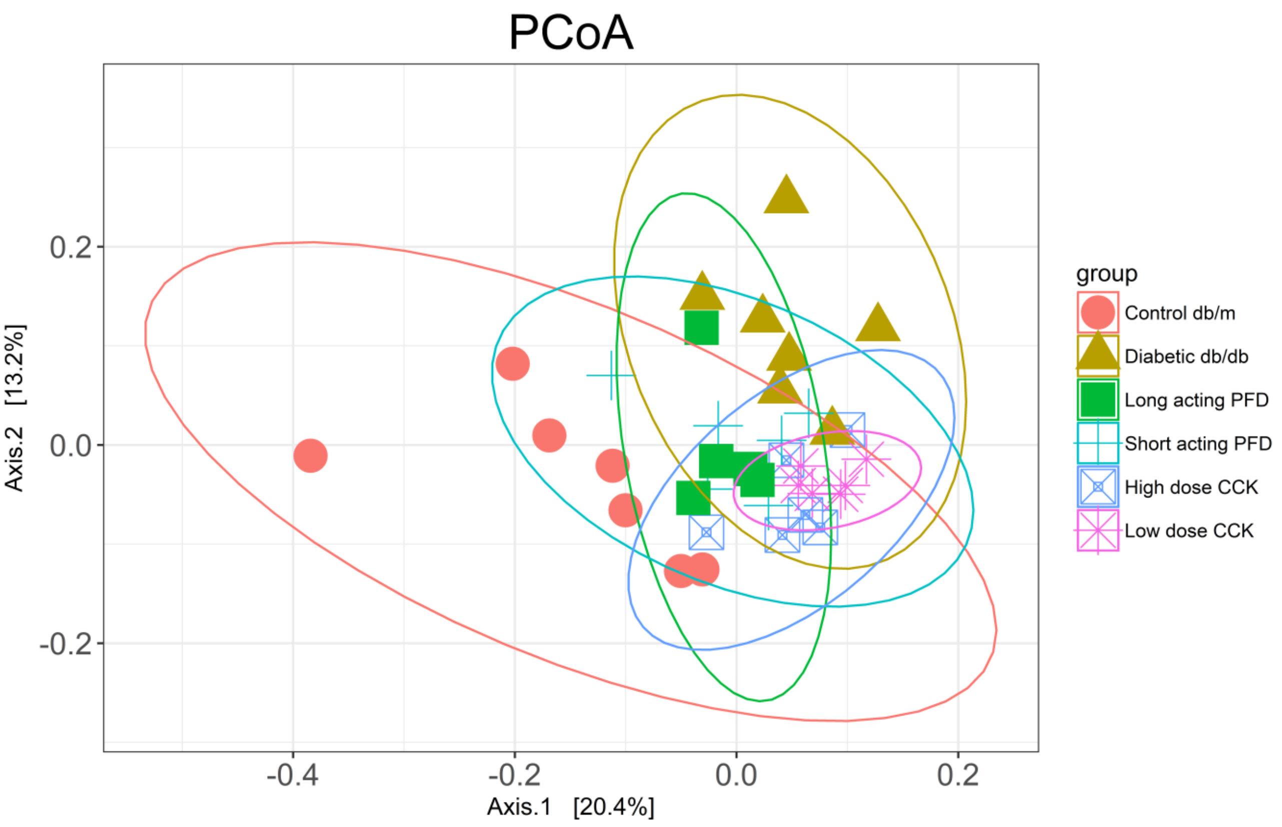

Supplementary Figure S2**.** Unweighted PCoA plot for all the groups along with 95% confidence interval.

Supplementary Table T1: Alpha diversity with Shannon and Chao1 diversity index differences using Wilcoxon test of the between control db/m and diabetic db/db and the four treated groups.

	Shannon Diversity Index		Chao1 Diversity Index	
	Control db/m	Diabetic db/db	Control db/m	Diabetic db/db
Diabetic db/db	0.03	NA	0.76	NA
Long acting PFD	0.27	0.07	0.94	0.22
Short acting PFD	0.06	1.00	0.95	0.22
High dose CCK	0.07	0.56	0.50	0.94
Low dose CCK	0.07	0.27	0.94	0.22

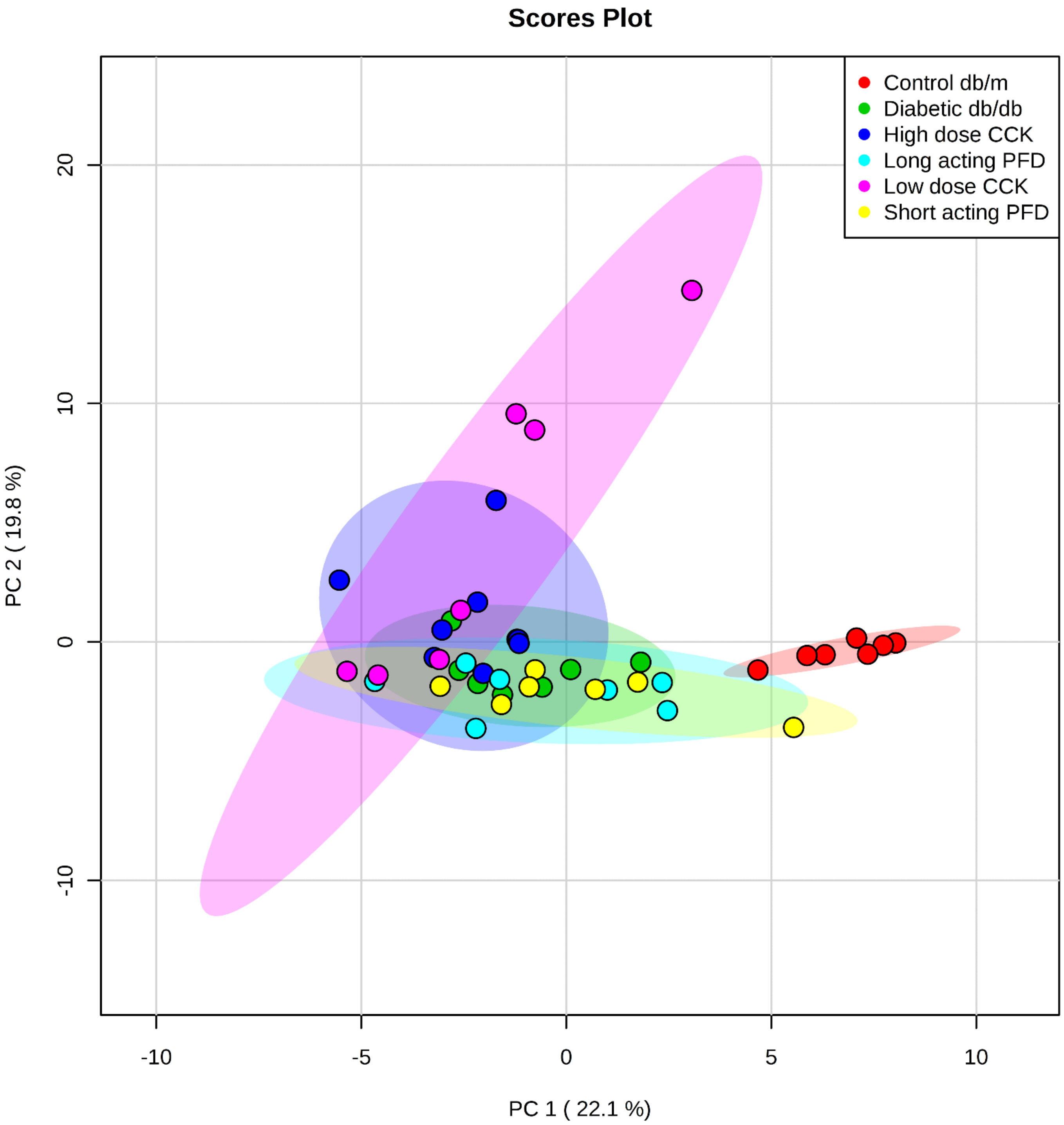

Supplementary Figure S3: PCA plot with ellipse denotes 95% confidence interval. This data was generated using 61 metabolites values from control db/m, diabetic db/db and the four drug-treated db/db mice.

Supplementary Table T2: Log fold change of various metabolites in diabetic db/db mice and four treated groups in comparison with control db/m mice.

	db/db mice		Long acting PFD		Short acting PFD		High dose CCK		Low dose CCK	
**Metabolite**	**FC**	**P.values**	**FC**	**P.values**	**FC**	**P.values**	**FC**	**P.values**	**FC**	**P.values**
3-Hydroxybutyric acid	8.5	0.01	8.0	0.01	8.8	0.00	7.8	0.00	8.4	0.03
Acetoacetic acid	8.3	0.03	7.2	0.02	8.7	0.01	7.2	0.03	8.6	0.09
2-Hydroxybutyric acid	3.8	0.07	9.4	0.33	2.0	0.40	3.7	0.01	6.5	0.12
3-keto-2-Methylbutyrate	3.7	0.03	2.9	0.01	4.0	0.00	2.6	0.04	4.1	0.11
3-Hydroxymethylglutaric acid	2.9	0.00	1.4	0.01			3.1	0.00	3.4	0.00
Glyceric acid	-2.7	0.00	-2.0	0.00	-2.2	0.00	-2.8	0.00	-2.3	0.00
Pyruvic acid	2.5	0.01	3.2	0.00	3.2	0.00	3.4	0.00	3.7	0.00
2-Ketobutyric acid	2.5	0.01	3.2	0.00	3.2	0.00	3.3	0.00	3.6	0.00
L-Lactic acid	2.4	0.00	2.7	0.04	2.6	0.02	4.1	0.00	4.5	0.00
2-Hydroxy-3-methylbutyric acid	2.3	0.01	2.1	0.07	1.9	0.05	3.6	0.00	4.1	0.00
Uracil	-2.3	0.00	-1.8	0.00	-1.6	0.00	-2.0	0.00	-1.7	0.00
2-Ethylhydracrylic acid	2.1	0.00	1.9	0.00	1.9	0.00	1.7	0.00	1.4	0.09
S-3-Hydroxyisobutyric acid	2.0	0.00	2.4	0.00	2.2	0.00	2.2	0.00	2.4	0.00
Palmitic acid	-1.7	0.00	-2.9	0.00	-2.2	0.00	-1.7	0.00	-3.3	0.00
Malonic acid	1.6	0.13	1.7	0.15	1.6	0.00				
Oxoglutaric acid	1.6	0.00	1.6	0.00	1.5	0.00	1.8	0.00	2.2	0.00
L-Octanoylcarnitine	-1.5	0.00	-1.1	0.02	-2.0	0.00	-1.7	0.00	-1.5	0.00
Benzoic acid	1.5	0.25	2.4	0.01			3.5	0.01	3.9	0.01
Fumaric acid	1.4	0.01			1.0	0.07	-1.2	0.00	-1.9	0.00
L-2-Hydroxyglutaric acid	-1.4	0.00								
Glutaric acid	-1.4	0.00	-1.1	0.00						
Leucinic acid	1.4	0.00	1.1	0.00	1.4	0.00	1.3	0.04	2.5	0.05
3-Hydroxyadipic acid	1.3	0.01					1.0	0.05		
Orotic acid	-1.3	0.00					-1.3	0.00		
Succinic acid	-1.2	0.00								
Hippuric acid	-1.2	0.00	-1.6	0.00	-2.1	0.00	-1.9	0.00		
Stearic acid	-1.2	0.00	-6.6	0.00	-6.4	0.00			-1.8	0.00
Alpha-ketoisovaleric acid	-1.2	0.00								
p-Hydroxyphenylacetic acid	-1.0	0.00	-1.9	0.00	-1.6	0.00	-1.8	0.00	-2.3	0.00
